# Assessment of radiation knowledge among medical personnel in nuclear emergency preparedness: a cross-sectional study

**DOI:** 10.3389/fpubh.2025.1547818

**Published:** 2025-02-26

**Authors:** Yanjun Xie, Xining Wang, Yuemin Lan, Xinyu Xu, Shaoteng Shi, Zhihao Yang, Hongqiu Li, Jing Han, Yulong Liu

**Affiliations:** ^1^The Second Afffliated Hospital of Soochow University, Suzhou, China; ^2^Global Health Institute, Health Science Center, Xi’an Jiaotong University, Xi’an, Shaanxi, China; ^3^Department of Occupational and Environmental Health, School of Public Health, Health Science Center, Xi’an Jiaotong University, Xi’an, Shaanxi, China; ^4^State Key Laboratory of Radiation Medicine and Protection, School of Radiation Medicine and Protection, Medical College of Soochow University, Suzhou, China

**Keywords:** medical personnel, radiation knowledge, nuclear emergency, awareness, preparedness

## Abstract

**Background:**

Radiation literacy, encompassing the understanding of basic principles, applications, risks, and protective measures related to ionizing radiation, is critical for medical personnel working in jobs that involve the use of radioactive materials or medical imaging. In the context of nuclear emergency preparedness, the level of radiation knowledge among healthcare professionals—such as doctors, nurses, and radiographers—directly influences the effectiveness and safety of emergency responses. This study aims to address this gap by evaluating the radiation knowledge of medical personnel and identifying areas for improvement in profession-specific training programs.

**Methods:**

A cross-sectional study was conducted using a convenience sampling method. The study included 723 participants attending a medical emergency response exercise and clinical management workshop on radiation injury in Suzhou, China, in November 2023. Data were collected through a structured questionnaire, descriptive statistics and chi-square tests were performed to analyze participants’ radiation knowledge and identify variations across different professional groups.

**Results:**

The majority of participants were female (64.73%), married (75.10%), and held an undergraduate degree (69.99%). Nurses (40.11%) and clinical doctors (30.29%) constituted the largest professional groups. Significant disparities in radiation knowledge were observed among healthcare workers. Nurses and management personnel demonstrated a stronger grasp of fundamental radiation concepts, such as radioactive nuclides and absorbed doses, compared to clinical doctors. For instance, 85.52% of nursing personnel and 72.34% of management personnel accurately identified the half-life of iodine-131, while only 49.32% of clinical doctors showed comparable knowledge. Furthermore, substantial differences in radiation emergency response capabilities were noted across professions. These findings emphasize the necessity for tailored, profession-specific training programs in radiation protection and emergency preparedness.

**Conclusion:**

The study reveals a generally insufficient understanding of basic radiation concepts and emergency response principles among medical personnel. Significant variations in radiation knowledge were observed across different professional groups, highlighting the need for specialized training modules. These modules should focus on fundamental radiation concepts, radiation exposure effects, and emergency response protocols, with content customized to address the unique needs of each professional group. By implementing such targeted training, the overall effectiveness and safety of nuclear emergency responses can be significantly enhanced.

## 1 Background

Radiation literacy is the understanding and knowledge of the basic principles, applications, risks and protective measures of ionizing radiation (1–3). Diagnostic and therapeutic procedures have been revolutionized with the use of nuclear technologies in the practice of medicine, providing remarkable accuracy and effectiveness (4, 5). However, while these technologies improve patient outcomes, they also pose potential risks to patients and healthcare professionals due to radiation exposure (6–8). Therefore, it is imperative that healthcare professionals have a thorough understanding of radiation safety principles in order to effectively minimize these risks.

Radiation healthcare professionals often work in jobs that involve the use of radioactive materials or medical imaging, where they are required to handle radioactive materials and equipment and are at risk of radiation exposure (9). China has issued multiple standards and technical specifications, such as the Basic Standards for Ionizing Radiation Protection and Radiation Source Safety (GB 18871-2002), which clarify the specific requirements and operational norms for radiation protection (10). Despite the critical importance of radiation safety, there is evidence to suggest that knowledge levels among healthcare professionals may be inadequate. The results of a study conducted showed that 63% of first responders had received training related to radiological terrorism, while only 50% of first responders had used personal protective equipment (PPE) in the past year (11). A research survey in China showed that clinical nurses scored 60.40 points in the nuclear emergency rescue knowledge test, with 0 scoring good or above, 52 scoring moderate (18.6%), 103 scoring passing (37.0%), and 124 scoring failing (44.4%) (12). These gaps in knowledge can lead to unsatisfactory safety measures and increase the likelihood of radiation injury (13). Therefore, there is a need to assess the current state of radiation knowledge among medical personnel, especially those who have received nuclear training, to identify areas for improvement.

In China, there are specific regulations and requirements for radiation protection in medical practice. According to the regulatory framework of the National Health and Wellness Commission of China, healthcare professionals working with ionizing radiation must obtain a radiation protection license, which requires completing an accredited training programme and passing a certification examination. These requirements are enforced by the National Health and Wellness Commission (NHWC), which sets radiation safety standards and ensures compliance through regular 2-yearly inspections and audits (14). Nuclear training courses are designed to equip medical personnel with the necessary skills and knowledge to handle and use radioactive materials safely. These programmes typically cover a range of topics including principles of radiation physics, radiation biology, radiation protection and regulatory requirements (15, 16). However, it is uncertain whether the local health department has implemented the relevant assessment requirements, and the effectiveness of these training programs in imparting sufficient radiation knowledge remains uncertain (17).

Although there have been many studies examining the level of knowledge and awareness of healthcare workers regarding radiation protection, these studies have tended to focus on specific professional groups or a single healthcare organization and have lacked a comprehensive investigation of the radiation knowledge of healthcare workers with different characteristics during their training for nuclear emergencies (18, 19). This study aims to fill this gap by covering healthcare workers with varying professional experiences across multiple regions. The novelty of this research lies in its broad scope and detailed analysis, which will provide a scientific foundation for developing more targeted radiation protection training programs, ultimately enhancing the preparedness and safety of medical staff in nuclear emergency situations.

## 2 Materials and methods

This study employed a cross-sectional survey design to evaluate healthcare workers’ knowledge and practices regarding radiation protection during a Medical Emergency Response Exercise and Clinical Management of Nuclear and Radiation Injuries workshop in November 2023. The survey targeted healthcare professionals involved in radiation-related occupations, while those who refused to participate or did not complete the questionnaire were excluded from the analysis.

A structured questionnaire was used for data collection, consisting of 47 questions divided into five main sections. These sections were as follows: Part 1, Demographic Characteristics (9 questions); Part 2, Basic Knowledge of Radiation (10 questions); Part 3, Effects of Radiation Exposure (10 questions); Part 4, Radiation Emergency Response Capabilities (10 questions); and Part 5, Radiation Medical Management (8 questions). Prior to full-scale implementation, the questionnaire was piloted to ensure clarity, reliability, and validity. The Cronbach’s alpha coefficient in this scale is 0.745, which is greater than 0.7, indicating high reliability in all dimensions of the scale (Table 1). The KMO value in this scale is 0.817, and if the KMO value is greater than 0.8, it meets the conditions for conducting factor analysis. The Bartlett sphericity test result shows an approximate chi square value of 2142.503. In addition, a significance probability *p* < 0.01 indicates that a significant level has been reached (Table 2).

**Table 1:** Scale reliability test.

Variables	Items	Cronbach’s
Basic knowledge of radiation	10	0.514
Effects of radiation exposure	10	0.491
Radiation emergency response capabilities	10	0.403
Radiation medical management	8	0.502
Cronbach’s	38	0.745

**Table 2:** KMO and Bartlett inspection.

KMO sampling suitability quantity		0.817
Bartlett sphericity test	Approximate chi square	4279.171
	Freedom	703
	Significance	*p* < 0.01

This study employed the Kendall sample estimation method with a sample size of 5–10 times the number of items in the scale. To minimize potential error, the sample size was expanded by 20%. The scale consisted of 47 items, and the sample size was 47*10/80% = 587.5. Ultimately, 723 healthcare professionals from a wide range of backgrounds, experience levels, and job roles participated in the survey. This included clinicians (219, 30.29%), medical technicians (120, 16.60%), nursing staff (290, 40.11%), and management staff (94, 13.00%).

Inclusion criteria encompassed healthcare professionals with radiation-related job roles, while exclusion criteria applied to those who opted out of the study or failed to complete the questionnaire in its entirety. Data were collected through a self-administered questionnaire distributed during the workshop. The completed questionnaires were uniformly numbered and entered into the dataset using EpiData 3.1 software to ensure data accuracy and consistency.

Statistical analysis was performed using SPSS version 27.0. A chi-square test was used to compare the percentage of correct answers across different job categories for each section of the questionnaire. Statistical significance was set at a threshold of *p* = 0.05.

## 3 Results

This study included 723 participants, the majority of whom were female (64.73%) and married (75.10%). The demographic details of all participants are provided in Table 3. Most participants had an undergraduate level of education (69.99%), with a smaller proportion holding graduate degrees (10.37%). Regarding job titles, junior (34.85%) and intermediate (35.27%) positions accounted for the largest proportion. In terms of professional roles, the nursing staff made up the largest group (40.11%), followed by clinical doctors (30.29%), with management personnel representing the smallest group (13.00%).

**Table 3:** Demographic features of participants (*n* = 723).

Variables	*N*	Percentage (%)
Sex		
Male	255	35.27%
Female	468	64.73%
Educational level		
Junior college and below	142	19.64%
Undergraduate	506	69.99%
Postgraduate	75	10.37%
Marital status		
Unmarried	180	24.90%
Married	543	75.10%
Professional title		
None of primary	76	10.51%
Junior	252	34.85%
Middle	255	35.27%
Senior	140	19.37%
Job post		
Clinician	219	30.29%
Medical technician	120	16.60%
Nursing	290	40.11%
Management	94	13.00%
Work in radiation medicine		
No	566	78.28%
Yes	157	21.72%
Participate in drills or training		
No	402	55.60%
Yes	321	44.40%
Participate in rescue operations		
No	555	76.76%
Yes	168	23.24%

Additionally, more than two-thirds of the respondents did not work in radiation medicine (78.28%) or participated in rescue operations (76.76%). However, a significant portion of the participants had taken part in exercises or training related to radiation medicine (44.40%) (Table 3).

**Figure 1:**
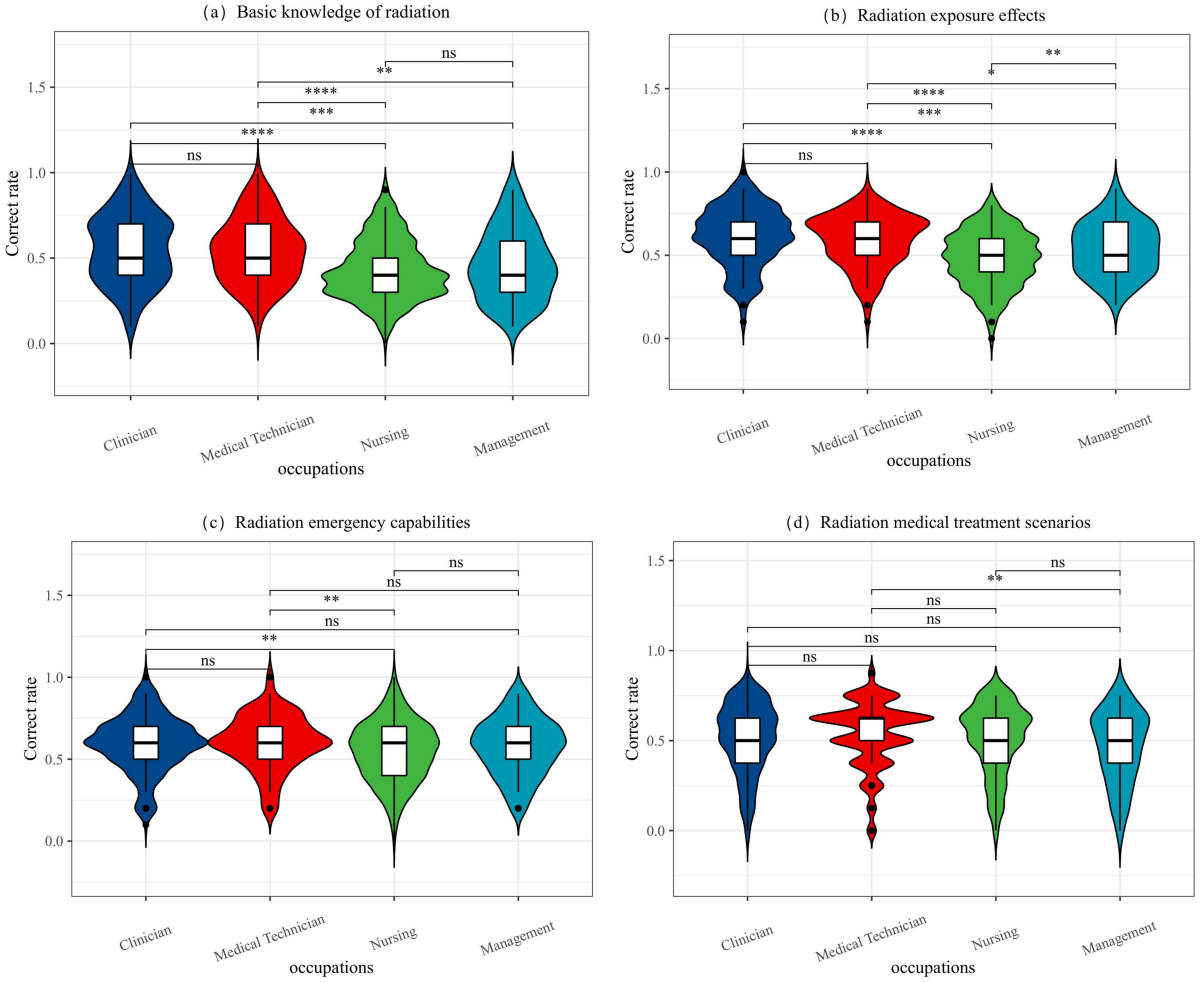
The difference between the correct rate of each module in 4 occupations. **(a)** Basic knowledge of radiation, **(b)** Radiation exposure effects, **(c)** Radiation emergency capabilities, **(d)** Radiation medical treatment scenarios. “*” means that the difference between the two labeled is statistically significant (*p* < 0.05); the greater the number of “*,” the smaller the *P*; “ns” means that the difference between the two labeled is not statistically significant (*p* > 0.05).

Table 4 and Figure 1 illustrate the differences in overall accuracy across the four knowledge modules among medical staff in different job types. The modules include basic knowledge of radiation, radiation exposure effects, radiation emergency capabilities, and radiation medical treatment scenarios. The average correct response rates for these modules were 49, 54, 57, and 51%, respectively. These results indicate that more than half of the medical workers lacked sufficient mastery of the fundamental knowledge related to radiation scenarios. In terms of individual knowledge modules, significant statistical differences were observed in the accuracy rates for basic knowledge of radiation, radiation exposure effects, and radiation emergency capabilities among medical personnel in different positions (*p* < 0.05). Specifically, management personnel demonstrated significantly lower accuracy rates compared to clinicians and nursing staff. However, in the module on radiation medical treatment scenarios, no significant differences were found in the correct response rates across the four occupational types, with all groups averaging around 50%.

**Table 4:** The correct rate of each module for the 4 occupations (Mean ± SD).

Modules	Total	Clinician	Medical technician	Nursing	Management	*p*
Basic knowledge of radiation	0.49 ± 0.20	0.54 ± 0.19	0.46 ± 0.20	0.55 ± 0.20	0.43 ± 0.18	<0.001
Radiation exposure effects	0.54 ± 0.18	0.59 ± 0.16	0.54 ± 0.16	0.61 ± 0.18	0.48 ± 0.17	<0.001
Radiation emergency capabilities	0.57 ± 0.18	0.60 ± 0.17	0.57 ± 0.17	0.59 ± 0.17	0.55 ± 0.18	0.005
Radiation medical treatment scenarios	0.51 ± 0.19	0.54 ± 0.18	0.46 ± 0.20	0.51 ± 0.19	0.51 ± 0.19	0.059

Table 5 highlights significant differences in basic radiation knowledge among different occupations. Nursing and management professionals demonstrated higher correct response rates on topics such as radioactive nuclides, absorbed dose, and the half-life of radioactive materials, with statistically significant differences (*p* < 0.05). While over half of the participants demonstrated an understanding of radiation hazards, clinical physicians had a relatively low correct response rate (49.32%, *p* < 0.001). Most nurses accurately identified the physical half-life of iodine-131 (85.52%, *p* < 0.001), whereas only a small proportion of clinical doctors correctly answered that photons are gamma rays and X-rays (22.83%, *p* = 0.029). Additionally, most medical technicians were able to correctly distinguish between nuclear emergencies and nuclear attacks (84.17%, *p* = 0.011), a difference that was statistically significant. Based on the data presented in Table 5, it is evident that nursing staff possess a relatively high level of radiation knowledge compared to other occupational groups.

**Table 5:** The basic knowledge of radiation for different occupations.

Variables	Clinician (*n* = 219)	Medical technician (*n* = 120)	Nursing (*n* = 290)	Management (*n* = 94)	*p*
	Percentage of correct answers				
A1. Radioactive nuclides are unstable atomic nuclei. (T)					
	59 (26.94%)	31 (25.83%)	109 (37.59%)	30 (31.91%)	0.031
A2. Radioactive decay activity is the decay rate of a radioactive isotope. (F)					
	34 (15.53%)	18 (15%)	64 (22.07%)	18 (19.15%)	0.193
A3. Absorbed dose measures the harm to biological tissue from radiation. (F)					
	108 (49.32%)	73 (60.83%)	200 (68.97%)	60 (63.83%)	*p* < 0.001
A4. The annual effective dose from natural background radiation is about two mSv. (T)					
	82 (37.44%)	33 (27.5%)	122 (42.07%)	37 (39.36%)	0.051
A5. The penetration distance of alpha particles in air is in centimeters. (T)					
	143 (65.3%)	75 (62.5%)	206 (71.03%)	68 (72.34%)	0.217
A6. The half-life of cobalt-60 (Co-60) is 74 days.(F)					
	109 (49.77%)	61 (50.83%)	172 (59.31%)	63 (67.02%)	0.014
A7. The physical half-life of iodine-131 is approximately 8 days.(T)					
	144 (65.75%)	73 (60.83%)	248 (85.52%)	68 (72.34%)	*p* < 0.001
A8. Photons are gamma rays and X-rays. (T)					
	50 (22.83%)	27 (22.5%)	79 (27.24%)	28 (29.79%)	0.429
A9. Radioactive isotopes produce X-rays. (F)					
	101 (46.12%)	56 (46.67%)	220 (75.86%)	61 (64.89%)	*p* < 0.001
A10. A nuclear emergency event is similar to a nuclear attack or war. (T)					
	153 (69.86%)	101 (84.17%)	231 (79.66%)	74 (78.72%)	0.011

Table 6 summarizes the understanding of the impact of radiation exposure on different professions. Nursing professionals consistently demonstrated high correct response rates, particularly in areas such as deterministic effects (18.28%, *p* < 0.001) and hematological syndrome (48.28%, *p* < 0.001). Both management and nursing professionals also achieved high scores in their understanding of the use of micronuclei for biological dose assessment, with correct response rates of 89.36 and 93.45%, respectively (*p* = 0.004). The importance of protecting infants and pregnant women from the risk of thyroid cancer was well recognized in each group, especially in nursing (87.59%, *p* < 0.001), and the differences were statistically significant (Table 6).

**Table 6:** The radiation exposure effects for different occupations.

Variables	Clinician (*n* = 219)	Medical technician (*n* = 120)	Nursing (*n* = 290)	Management (*n* = 94)	*p*
	Percentage of correct answers				
B1. Deterministic effects: dose–response and threshold relationship. (T)					
	16 (7.31%)	14 (11.67%)	53 (18.28%)	7 (7.45%)	*p* < 0.001
B2. A dose–response relationship without a threshold characterizes random effects. (T)					
	43 (19.63%)	28 (23.33%)	93 (32.07%)	24 (25.53%)	0.014
B3. The advantage of using micronuclei to estimate biological dosages is the ease of analysis. (T)					
	186 (84.93%)	100 (83.33%)	271 (93.45%)	84 (89.36%)	0.004
B4. Hematologic syndromes occur at doses <2 Gy.(F)					
	52 (23.74%)	38 (31.67%)	140 (48.28%)	37 (39.36%)	*p* < 0.001
B5. Radioactive isotope contamination wounds are usually on the face. (F)					
	83 (37.9%)	38 (31.67%)	151 (52.07%)	35 (37.23%)	*p* < 0.001
B6. The organ that accumulates radioactive iodine is the thyroid. (T)					
	22 (10.05%)	12 (10%)	83 (28.62%)	13 (13.83%)	*p* < 0.001
B7. Infants and pregnant women should prioritize shielding from thyroid cancer risks due to radioactive iodine. (T)					
	161 (73.52%)	92 (76.67%)	254 (87.59%)	72 (76.60%)	*p* < 0.001
B8. The harmful effects of indoor pollution stem from both chemical and radiological sources. (T)					
	56 (25.57%)	37 (30.83%)	114 (39.31%)	47 (50%)	*p* < 0.001
B9. Biological effects of external exposure result from radioactive nuclides’ chemical and radiological toxicity. (F)					
	176 (80.37%)	107 (89.17%)	251 (86.55%)	74 (78.72%)	0.048
B10. The rise in childhood thyroid cancer post-Chornobyl in evacuated areas is due to short-lived radioactive iodine. (T)					
	59 (26.94%)	29 (24.17%)	103 (35.52%)	41 (43.62%)	0.004

Table 7 highlights significant differences in radiation emergency knowledge among various professions. Nursing professionals recorded the highest correct response rates for radiation protection principles (35.86%) and the prevention of deterministic effects (16.55%), with statistically significant differences (*p* = 0.002 and *p* = 0.023, respectively). Management professionals demonstrated the highest understanding of international nuclear event levels, achieving a correct response rate of 50%, which was also statistically significant (*p* = 0.024). Clinicians led in their knowledge of the National Nuclear Emergency Medical Rescue Team’s rapid response and self-sufficiency, with a correct response rate of 65.75% (*p* = 0.031). These findings emphasize the variability in radiation emergency knowledge across professions and highlight the need for targeted training programs to address specific knowledge gaps among different occupational groups.

**Table 7:** The radiation emergency capabilities of different occupations.

Variables	Clinician (*n* = 219)	Medical technician (*n* = 120)	Nursing (*n* = 290)	Management (*n* = 94)	*p*
	Percentage of correct answers				
C1. The three principles of radiation protection are shielding, notification, and dose limitations. (F)					
	54 (24.66%)	22 (18.33%)	104 (35.86%)	27 (28.72%)	0.002
C2. Iodine tablets are best taken one hour before exposure to radioactive iodine. (T)					
	175 (79.91%)	99 (82.5%)	246 (84.83%)	76 (80.85%)	0.517
C3. The main goal of nuclear and radiation protection is to prevent deterministic effects. (T)					
	20 (9.13%)	9 (7.5%)	48 (16.55%)	11 (11.7%)	0.023
C4. After a nuclear accident, prioritize saving lives and controlling contaminant spread. (T)					
	40 (18.26%)	33 (27.5%)	69 (23.79%)	24 (25.53%)	0.204
C5. International Nuclear events are categorized into six levels. (F)					
	81 (36.99%)	51 (42.5%)	144 (49.66%)	47 (50%)	0.024
C6. The National Nuclear Emergency Medical Rescue Team requires rapid response, analysis, and self-sufficiency. (T)					
	144 (65.75%)	65 (54.17%)	183 (63.1%)	48 (51.06%)	0.031
C7. Key concerns for rescue teams include reliance on part-time responders and a lack of nuclear physics expertise. (T)					
	69 (31.51%)	30 (25%)	108 (37.24%)	34 (36.17%)	0.093
C8. The basic requirements for nuclear emergency training include adhering to guidelines and developing plans. (T)					
	50 (22.83%)	21 (17.5%)	65 (22.41%)	22 (23.4%)	0.650
C9. A rapid response necessitates deployment within one hour of receiving orders.(F)					
	137 (62.56%)	74 (61.67%)	165 (56.9%)	59 (62.77%)	0.533
C10. Nuclear emergency rescue requires managing teams, assessing accidents, and proposing solutions. (T)					
	118 (53.88%)	74 (61.67%)	183 (63.1%)	53 (56.38%)	0.171

Table 8 shows the response levels of different professions regarding radiation medicine. Significant differences were observed in the knowledge related to high-dose local radiation therapy, with management and nursing professionals achieving the highest accuracy rates of 36.17 and 35.17%, respectively, and these differences were statistically significant (*p* < 0.001). In addition, a notable difference was observed in the understanding of the management of radiation medicine in second-level and higher hospitals, with management professionals scoring the highest at 53.19% (*p* = 0.032). However, other areas, such as prioritizing accident data collection and the role of hospital nursing and burn departments, did not show statistically significant differences. These results highlight variations in radiation rescue knowledge across different professions and underscore the need for targeted training in other occupational groups, as summarized in Table 8.

**Table 8:** The radiation medical treatment scenarios for different occupations.

Variables	Clinician (*n* = 219)	Medical technician (*n* = 120)	Nursing (*n* = 290)	Management (*n* = 94)	*p*
	Percentage of correct answers				
D1. Treatments for high-dose localized radiation include skin grafts and stem cell therapy. (T)					
	35 (15.98%)	22 (18.33%)	102 (35.17%)	34 (36.17%)	*p* < 0.001
D2. Prioritize collecting accident data in radiation accidents. (F)					
	217 (99.09%)	116 (96.67%)	278 (95.86%)	93 (98.94%)	0.101
D3. Is it true that nuclear events commonly associate with explosions, radiation burns, and respiratory injuries?(T)					
	78 (35.62%)	38 (31.67%)	88 (30.34%)	35 (37.23%)	0.483
D4. Second-level and above hospitals should manage radiation medical care. (T)					
	96 (43.84%)	41 (34.17%)	115 (39.66%)	50 (53.19%)	0.032
D5. The hospital’s care and burn departments are crucial in nuclear rescue. (T)					
	72 (32.88%)	38 (31.67%)	93 (32.07%)	40 (42.55%)	0.269
D6. Clinical radiology professionals manage radioactive materials in accidents. (T)					
	92 (42.01%)	54 (45%)	129 (44.48%)	35 (37.23%)	0.612
D7. Surgeons need to master skills such as first aid and radiation damage. (T)					
	47 (21.46%)	17 (14.17%)	55 (18.97%)	25 (26.6%)	0.132
D8. Nurses must adeptly use testing equipment to detect surface contamination. (F)					
	217 (99.09%)	120 (100%)	283 (97.59%)	91 (96.81%)	0.162

To sum up, our research has demonstrated that nursing and management personnel exhibit greater accuracy in basic radiation knowledge compared to clinical doctors and medical technicians, who tend to have lower accuracy rates. These findings highlight the disparities in radiation knowledge among various occupational groups and underscore the necessity for targeted training to enhance overall radiation response capabilities.

## 4 Discussion

This study evaluated the radiation knowledge level of 723 medical personnel with different types of jobs, professional titles, educational backgrounds, and training experiences through a cross-sectional investigation. The results showed that medical personnel with different background characteristics had significant differences in basic radiation knowledge, radiation exposure effects, emergency response-ability, and medical treatment ability. This discovery prompts us to re-examine the effectiveness of current nuclear emergency preparedness training, particularly in addressing the training needs and knowledge gaps of medical personnel in different roles.

In terms of basic radiation knowledge, although most medical personnel have a sure grasp of practical knowledge such as “absorbed dose” and “*α* particle penetration distance, “the cognition degree of basic concepts such as “radionuclide” and “radioactivity” is generally not high, and the accuracy rate of clinicians and medical technicians is less than 30%. These differences not only reflect knowledge gaps between jobs and degrees, but also highlight the need to design educational content for different medical roles. This is consistent with the findings of Alghamdi et al. (20), who found that healthcare professionals have a woefully inadequate understanding of the concept of radiation and its implications. Similarly, Kew et al. (21) also found through a questionnaire survey that there was a noticeable gap in the theoretical knowledge of radiation protection among clinicians. These findings suggest the need to enhance the foundational theory of radiation training for medical staff. It is important to develop clear learning objectives tailored to the specific responsibilities of different medical roles. For example, clinicians may need to learn more about radiation protection and radiobiology, while paramedics may need to learn more about emergency management (22).

In terms of the cognition of radiation exposure effect, medical personnel in different positions showed significant differences in their grasp of the concepts of “deterministic effect, “random effect,” and “radioactive iodine accumulation organ,” The demand for knowledge of these concepts is closely related to their daily work, indicating that different positions require different scope of knowledge. As Hendee et al. (23) said, clinicians need a systematic study of radiation biology, and it is difficult to fully understand the potential harm of radiation to the human body. Clinicians, nurses, and medical technicians have a relatively good grasp of practical knowledge such as “micronucleus dose estimation” and “blood syndrome onset dose,” which may be related to their more practical duties. Managers have the highest awareness of “internal pollution damage effects,” possibly because they need to control and judge these conditions. Therefore, when medical personnel in different positions receive radiation protection training, the breadth and depth of knowledge should be customized according to their work content (10).

In terms of nuclear emergency response ability, different types of work have a distinct grasp of basic knowledge such as “radiation protection principles,” “accident classification,” and “emergency capacity requirements.” However, all types of workers generally have low awareness of specific practical aspects such as “emergency response objectives,” “primary tasks,” and “emergency exercise requirements,” This finding aligns with the research conducted by Bushberg et al. (24) and Obrador (25), who reported that medical personnel often lack practical skills training related to nuclear and radiation emergency response. In the future, it is necessary to strengthen not only basic theoretical training but also actual combat exercises to improve the rapid response and emergency response capabilities of medical personnel.

In terms of radiation medicine processing ability, the cognition level of management and nursing staff on “on-site medical management” and “hospital division of labor” was significantly higher than that of other jobs, but the cognition level of corresponding jobs on “clinical ability needs” and “surgical ability needs” was at a low level. This situation is alarming and in line with the views of Chen et al. (26), that is, medical personnel of different levels and professional backgrounds have significant differences in professional knowledge and skills and need to be taught by classification. Clinical front-line personnel also need to improve in radiation-related medical treatment knowledge, possibly due to less exposure to such knowledge in their daily work (27). Therefore, in the future, attention should be paid to cultivating interdisciplinary talents and providing more emergency drill opportunities for medical personnel.

In summary, this study reveals critical gaps in the understanding of basic radiation concepts and emergency response principles among medical personnel, particularly among front-line clinical staff and those with lower educational attainment. These findings align with established scientific theories, such as the Knowledge Gap Hypothesis and Adult Learning Theory (28, 29), which emphasize the importance of tailored educational interventions to address varying levels of prior knowledge and professional roles. The results underscore the necessity for the Nuclear Emergency Response Agency to design and implement specialized training modules that focus on fundamental radiation concepts, radiation exposure effects, and emergency response protocols. Furthermore, targeted training programs should be developed to address the distinct knowledge and skill gaps identified across different medical roles, such as clinicians, nurses, and management personnel. Additionally, interdisciplinary training in radiation biology and radiation injury first aid should be integrated into existing curricula to enhance the overall quality of nuclear emergency medical rescue. By adopting these evidence-based strategies, the preparedness and effectiveness of medical personnel in nuclear emergencies can be significantly improved.

However, this study also has some limitations. Its cross-sectional design restricts the ability to assess changes in knowledge and skills over time, and self-reported data may introduce bias, as respondents might overestimate their knowledge or abilities. Additionally, while the study sample was diverse, it may not fully represent all healthcare settings or regions, which could affect the generalization of the findings. To address these limitations, future research should consider employing longitudinal designs and conducting objective evaluations whenever feasible to enhance the reliability of the results. Expanding the sample size and including a broader range of settings will also contribute to improving the generalization of the findings.

## Data Availability

The raw data supporting the conclusions of this article will be made available by the authors, without undue reservation.
